# Outcomes of Degenerative Cervical Myelopathy From The Perspective of Persons Living With the Condition: Findings of a Semistructured Interview Process With Partnered Internet Survey

**DOI:** 10.1177/2192568220953811

**Published:** 2020-11-18

**Authors:** Benjamin M. Davies, Colin Munro, Danyal Z. Khan, Siobhan M. Fitzpatrick, Bryn Hilton, Oliver D. Mowforth, Angus G. K. McNair, Iwan Sadler, Mark R. N. Kotter

**Affiliations:** 1 Division of Neurosurgery, Department of Clinical Neurosciences, University of Cambridge, Cambridge, UK; 2 Department of Psychology, University of Warwick, Coventry, UK; 3 Colchester Hospital University, East Suffolk and North Essex NHS Foundation Trust, Colchester, UK; 4 Bristol Medical School: Population Health Sciences, University of Bristol, Bristol, UK; 5 Myelopathy Support, Myelopathy.org, Cambridge, UK; 6 WT MRC Cambridge Stem Cell Institute, Anne McLaren Laboratory, Cambridge, UK

**Keywords:** cervical myelopathy, spondylosis, qualitative, thematic analysis, core outcomes set, consensus, patient perspectives

## Abstract

**Study Design:**

Mixed-methods cross-sectional study.

**Objectives:**

Degenerative cervical myelopathy (DCM) is a common and disabling condition. While classically, assessment and diagnosis has focused on neuromuscular symptoms, many other disabilities have been linked. The aim of this study was to explore the consequences of DCM for those with lived experience, producing a long list to inform the development of a core outcome set for DCM research.

**Methods:**

A 2-stage process was used: a focus group session of people with DCM (PwCM) and their supporters (n = 8) discussed the impact of DCM. This was used to develop a preliminary list of consequences, which were then placed into a survey of an online community of DCM sufferers (n = 224). Survey participants were asked to tick the consequences that they had experienced and given the opportunity to submit additional. Additional consequences were reviewed by a group of healthcare professionals and PwCM and included if not already represented. Demographic information including disease severity, age, and sex were captured for sampling comparison.

**Results:**

A total of 52 outcomes were identified from the focus group and nominally divided into 2 categories; symptoms (36 outcomes) and handicaps (18 outcomes), and further evaluated using a survey. All outcomes were recognized by at least 5% of respondents. A further 16 outcomes were added following the survey.

**Conclusions:**

A list of DCM consequences has been defined from the perspective of PwCM. This will now be evaluated as part of AO Spine RECODE-DCM, an international multistakeholder collaboration to establish a core outcome set for research.

## Background

Degenerative cervical myelopathy (DCM) is an umbrella term for injury of the cervical spinal cord due to degenerative changes of the surrounding spinal structures.^1^ It is the most common cause of spinal cord dysfunction worldwide, with estimated prevalence as high as 2% in adults.^2^

As the conduit between brain and body, disease of the cervical spinal cord can trigger a number of different symptoms. Classically DCM assessment has focused on neuromuscular function of the hands, arms, and legs, alongside bladder dysfunction.^3-5^ These domains, for example, are all measured by the modified Japanese Orthopedic Association scale, the international standard on disease severity and reference for treatment decision making.^6^ However, there are a number of reports of broader and prevalent disability,^7^ including headaches,^8^ movement disorders,^9^ respiratory dysfunction,^10^ and hypertension.^11^ A screening tool developed in Japan identified chest pain as predictive of DCM.^12^

It is noted that in our recent evaluation of artificial intelligence, symptom checkers in the screening for DCM, we synthesized a number of narrative reviews on the diagnosis of myelopathy to generate a list of signs and symptoms. However, these articles did not universally overlap in their description.^7^

Taken together, these findings question whether our definition of what symptoms are associated with DCM is currently too narrow. This is a timely question to consider on a number of fronts.

First, time to diagnosis and treatment has been identified as one of the few modifiable factors for improving treatment response in DCM: analysis of the AO Spine datasets initially identified <6 months as significant,^13^ and this has recently been further refined to <4 months.^14^ Unfortunately, most wait on average 2 to 5 years for treatment^15,16^ and many are never diagnosed.^1,2^ Efforts to target earlier diagnosis will benefit from a comprehensive understanding of potential signs and symptoms, especially at an early stage.

Second, AO Spine RECODE-DCM (aospine.org/recode), an international multistakeholder consensus process, has been established to develop recommendations to improve research efficiency in DCM.^17,18^ This includes the formation of a core outcome set [COS], to standardize assessment and reporting in research by defining the outcomes that should be reported as a minimum. COS development starts with the development of a “long list” of outcomes, which is put through a consensus process, to decide which outcomes are most important or “Core.” The formation of the “long list” of outcomes has been established by a variety of means, including systematic review of outcome reporting, domain mapping of outcome tools, and stakeholder interviews or surveys.^19^ While professional practice is well represented through systematic review, this may not represent the views of persons with DCM.^5,20^

The objective of this study therefore was to explore the consequences of DCM with people living with DCM. This would be used, alongside systematic reviews of medical literature, to produce a “long list” of outcomes in DCM that would be refined through a consensus process as part of AO Spine RECODE-DCM.

## Methods

**Figure 1.**
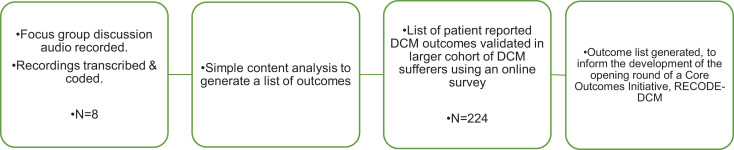
Flow diagram proving an overview of the methodological process. Workshops with PwCM and carers was used to develop a list of DCM effects. This were then processed by authors, including an individual suffering from DCM, and placed into an internet survey for DCM sufferers, to capture wider perspective. The findings of this, including any additional suggestions was then processed and used to form a long list of DCM outcomes. DCM, degenerative cervical myelopathy; PwCM, people with DCM.

A 2-stage process was undertaken (Figure 1): A focus group of persons with DCM (PwCM)^21^ and their supporters participated in a semistructured discussion on the impact of DCM, which informed a survey of an online community of DCM sufferers. The survey was granted ethical approval by the University of Cambridge Human Biology Research Ethics Committee.

### Semistructured Interviews

Attendees of a Patient and Public Involvement Day at Cambridge University Hospital on September 21, 2017 hosted by Myelopathy.org, a charity and support group for persons with DCM, were invited to participate in a focus group. Convenience sampling was employed, with the event advertised to registered members of Myelopathy.org. Participants were not known to researchers beforehand. Participants were asked to declare their age and gender. Attendees underwent a neurological assessment by a neurosurgeon, including calculation of their modified Japanese Orthopedic Association score (mJOA), the international standard for disease severity.^6^

Previous qualitative work for outcome projects has identified that participants have difficulties with the term “outcome.”^19^ Therefore prior to starting, alongside an outline of the current research program and the goals of the session, a group task was undertaken to list the potential effects of taking a train journey.

The sessions were facilitated by 2 interviewers (BMD and MRNK), both spinal surgeons and researchers experienced in the field of DCM. The groups were initially interviewed separately as “PwCM” and “Carers.” Interviewers aimed to cover domains identified from the research literature (function, pain, and quality of life),^5^ the subcategories of the international standard for DCM assessment, the mJOA (sensation, walking, bladder function, hand strength),^22^ and “less known symptoms.”^7^ Workshops were initiated through an open question *How does DCM affect you?* This was to enable both the establishment of a list of outcomes (as discussed in this article) and to enable thematic analysis (presented in a separate article), to provide context, significance, and intricacies of outcomes to support the establishment of domains.

Potential outcomes were highlighted by the facilitators during the workshops, and if the group was in agreement, noted on a paper card, placed in the center of the group akin to a Word Cloud. These were used to stimulate further discussion and to allow carers to view PwCM responses and vice versa. Following separate workshops, the groups were combined and further opportunity given to add additional outcomes or perspective on outcomes. This then led onto an outcome grouping exercise, which is the subject of a separate article. In total, the 3 sessions ran for 40 minutes. The sessions were audio-recorded and transcribed by 2 authors (DZK and SMF) with any discrepancies settled by discussion and mutual agreement. Outcomes identified on paper cards at the time were used to generate a list of outcomes. These were reviewed by BMD, CM, and IS to remove identical outcomes and group terms into common categories, in order to identify overlapping outcomes, which could be combined. For any outcomes with ambiguous terminology, investigators referred back to the audio transcripts for context, and adapted the wording at their discretion. All changes were made by mutual agreement. This revised outcome list was externally validated using an online survey.

### Online Survey

An internet survey was created using SurveyMonkey and is reported according reported according to the Checklist for Reporting Results of Internet E-Surveys (CHERRIES).^23^ Ethical approval was granted by the University of Cambridge. Participants were initially provided with an overview of the study and definition of DCM. By continuing into the survey, participants were confirming their diagnosis of DCM and providing consent to participate. A series of initial questions was used to provide sampling characteristics, including age, gender, history of surgical treatment, length of symptoms and disease severity, as measured using the self-reported mJOA score (p-mJOA).^24^ Respondents were then asked to tick which of the outcomes they had experienced and given the option to submit additional outcomes. Participants from the interview stage were contacted and specifically asked not to participate in this survey.

The survey was advertised to an online community of DCM sufferers, hosted by Myelopathy.org, an international charity for DCM. Advertisement was specifically made through 2 email calls, blog features on Myelopathy.org, and shared posts in Myelopathy Support, the peer-to-peer support community of Myelopathy.org hosted on Facebook. This community has previously been used to support online survey initiatives in DCM.^25^ The community is known to include a mix of pre- and postsurgical treatment sufferers, with disease demographics in keeping with the published literature. The only significant difference of note is the community has a higher proportion of female sufferers, as is typical of e-health support groups.

There was no incentive offered for participation in the survey. Once completed, survey respondents could not edit their results. IP addresses were tracked to prevent duplicate entries and allow users to return to the survey where they left off.

Descriptive statistics were used to synthesize sampling data and the demographics of those experiencing them. Outcomes with a prevalence of greater than 5% were carried forward into the final outcomes list. Outcomes with a prevalence of less than 5% or submitted as additional were reviewed by BMD, IS, and CM to establish generalizability to DCM. For additional submissions, this involved the independent processing of outcomes as either out of scope, already represented or new. Results were combined, and discrepancies settled by mutual agreement. Unrepresented outcomes were processed as previous.

## Results

### Interview Phase

Eight individuals participated in the semistructured interviews, including 5 PwCM (3 men and 2 women) and 3 supporters (all women, 2 identifying as partners and 1 as a close friend). The average age of PwCM was 53years. Four PwCM had undergone surgery for DCM, 3 within the past 2 years, and 1 over 2 years ago. One patient was awaiting surgery. All attendees identified as White Caucasian. The median mJOA of 11 (±interquartile range [IQR] 2), indicating these PwCM had moderate to severe DCM. The workshops generated 58, discretely recorded effects. PwCM provided more (54, 93%) than carers (17, 29%). Carers identified 4 problems not reported by PwCM; difficulty initiating urination, loss of coordination, inability to make plans, and altered cognition (Supporting Information 1, see Supplementary Material). The combining of groups did not generate additional suggestions. These findings were processed as outlined, and a shortlist of 52 outcomes placed into an internet survey (Supporting Information 2, see Supplementary Material).

### Online Survey Phase

The list of outcomes generated from patient and carer interviews were processed by investigators, to generate a list of 52 outcomes. These were nominally divided into 2 categories; symptoms (36 outcomes) and handicaps (18 outcomes), to break up the survey list.

The survey ran from January 2017 until November 2017. The survey was accessed 294 times, including 8 duplicate entries and 62 incomplete entries. Therefore 224 responses underwent analysis. The respondents were on average 56.6 years old, lived with DCM for 8.2 years, and had a mJOA of 11.6. Respondents were more likely to be female (76%) and undergone surgery (62%).

**Figure 2.**
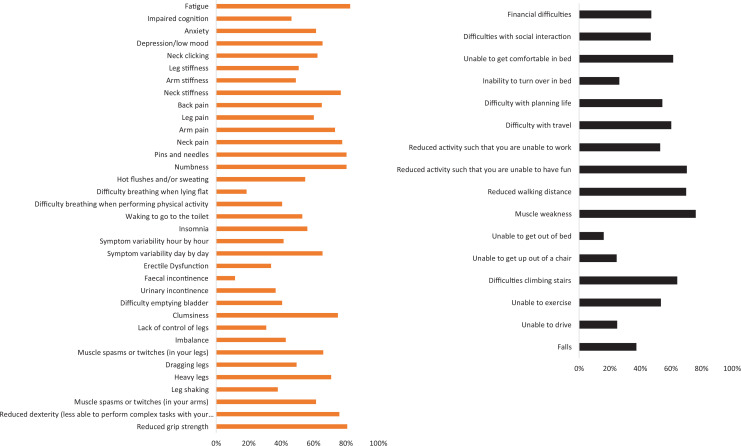
Proportion of survey respondents expiring a listed symptom (orange bar chart) or effect on life (black bar chart). All outcomes were experienced by at least 5% of surveyed individuals (vertical blue line).

All 52 outcomes passed the predefined 5% threshold for review (Figure 2). For internal consistency, erectile dysfunction was not reported by female respondents. It was instead reported by 34% of male respondents. Otherwise, there was no difference in gender, surgical history, mJOA, or timed lived with DCM between those who did and did not report experience an outcome (Supporting Information 4, see Supplementary Material).

A total of 94 (42%) respondents submitted additional outcomes (Supporting Information 3, see Supplementary Material); 80 reporting additional symptoms and 52 additional effects on life. Thirty-eight of these respondents submitted additional information in both categories. In general, respondents submitted multiple additional outcomes. The majority were felt to already be represented. Noteworthy omissions included headache (15 references), dizziness (10 references), burning dysesthesia (10 references), stabbing/electrical shock sensation (5 references), sexual dysfunction (6 references), altered hearing (2 references), and altered vision (7 references) (Table 1).

**Table 1.** Outcomes (Symptoms) Derived From Open Text questions, Including Number of Citations and the Demographics of Respondents Submitting.

Outcome	Number respondents suggested	For those experiencing an outcome			
		mJOA, mean ± SD	Female, %	Undergone surgery, %	Time lived with DCM, years, mean ± SD
Headache	15	10.3 ± 1.9	87	73	10.5 ± 7.4
Dizziness	10	12.4 ± 2.7	90	70	14.3 ± 16.7
Burning	10	11.0 ± 2.4	80	60	6.5 ± 3.6
Stabbing/electrical shock (Lhermitte sign)	5	13.4 ± 2.9	60	80	6.9 ± 2.9
Sexual dysfunction	6	11.8 ± 1.3	83	100	6.4 ± 3.0
Hearing	2	8.5 ± 0.5	100	100	10.3 ± 5.8
Vision	7	11.0 ± 4.0	86	57	10.8 ± 12.1
Shoulder	6	11.3 ± 2.4	83	50	14.7 ± 8.7
Face	7	11.1 ± 1.5	71	86	10.5 ± 6.2

Abbreviations: mJOA, modified Japanese Orthopedic Association score; DCM, degenerative cervical myelopathy.

It should be noted that on initial review both hearing and visual impairments were felt to not be directly related to DCM. However, due to the number of submissions and on the basis of inclusivity and given published literature was identified citing similar observations in cervical spondylosis,^26^ these outcomes were long-listed. An additional theme identified was the desire to localize symptoms, particular sensory or pain symptoms beyond upper and lower limb, including specific reference to the shoulder and neck.

On this basis, the final long lists in Tables 2 and Table 3 were established. Outcomes that were included from the open-ended survey questions are marked in red, in order to distinguish them from those that have been answered by the total survey population.

**Table 2.** Final “Long List” of Patient-Reported Outcomes in Degenerative Cervical Myelopathy (DCM), Relating to Direct Symptoms.^a^

Long list of patient-reported outcomes in DCM (symptoms)	
Domain	Location
**Motor**	
Grip Strength	
Arm Strength	
Leg Strength	
Clumsiness	
Lack of control of legs	
Falls	
Loss of dexterity	
Imbalance	
Cramps, spasms, or twitches - at arms or legs	
Shaking (or tremor) - at arms or legs	
**Sensory**	
Pain	Neck
Numbness	Shoulder
Burning	Arm
Pins and needles/parasthesiae	Hand
Stiffness	Leg
Heaviness	Face
Headache	
Lhermitz phenomena/electrical stabbing shocks	
Hot flushes/temperature dysregulation/sweating	
Neck clicking	
Back pain	
Allodynia	
**Genitourinal/gastrointestinal**	
Erectile dysfunction	
Sexual dysfunction	
Difficulty emptying bladder	
Urinary incontinence	
Nausea and vomiting	
Swallowing difficulties/choking	
Constipation	
Fecal incontinence	
Abdominal pain	
**Miscellaneous**	
Difficulty breathing, when performing physical activity	
Difficulty breathing, when lying flat	
Insomnia	
Symptom variability	
Fatigue	
Impaired cognition	
Depression/low mood	
Anxiety	
Dizziness	
Eyesight problems	
Tinnitus	
Hearing impairment	

^a^ A number of symptoms could be experienced in different locations, and this is referenced by the connecting brackets, including the perception or perceptions and the suggestion locations. Some symptoms were taken from the open-ended survey question and have therefore not been explored across the entire survey population (in red).

**Table 3.** Long List of Patient-Reported Outcomes in Degenerative Cervical Myelopathy (DCM), Relating to Life Effects, Referred to as the Handicap.^a^

Long list of patient-reported outcomes in DCM (handicaps)
Unable to drive
Unable to exercise
Difficulty climbing stairs
Unable to get out of chair
Unable to get out of bed
Unable to roll of in bed
Unable to get comfortable in bed
Reduced walking distance
Reduced activity that you are unable to have fun
Reduced activity such that you are unable to work
Difficulty with travel
Difficulty with life planning
Difficulties with social interaction
Financial difficulties
Difficulty parenting, and in family life
Reduced sex life
Difficulty thinking, concentrating, or remembering things
Fear of recurrent disease, or deterioration following trauma

^a^Some handicaps were taken from the open-ended survey question and have therefore not been explored across the entire survey population (in red).

## Discussion

This is the first study to consider the effects of DCM from the perspective of those living with the condition. The described outcomes go far beyond our current “textbook” description of the disease syndrome and the research assessments in common usage.^5,7^ It is also unique in using their own words to articulate outcomes and providing an indication of symptom prevalence.

There are a number of noteworthy findings from this process. First, the recognition of female sexual dysfunction and of the different domains of sensation (eg, burning, paresthesia, temperature). Second, the prevalence and impact of symptom variability and sleep disturbance. Third, the experience of gastrointestinal and respiratory dysfunction. Fourth, the experience and prevalence of dyskinesias. Finally, the reporting of symptoms beyond our conventional framework, including headache, dizziness, visual, and auditory dysfunction.

Some of these experiences are not easily reconciled with our conventional understanding of the pathophysiology of DCM. It should be noted that the overall aim of this study was to produce a long list of patient-reported outcomes of DCM, without predefined bias. This is therefore an inclusive list, to inform future work. It should be interpreted in the context of its study design and wider literature, as discussed below.

### Limitations

Respondents belonged to a self-selecting group of individuals, recruited from an online community, who were asked to confirm they had received a diagnosis of DCM by a medical professional, after being presented with an explanation of the disease for verification purposes. While interview participants underwent clinical evaluation, no additional assessment was carried out on survey participants. It is possible therefore that some respondents did not have DCM and was also possible participants had coexistent health conditions, with disability misappropriated to DCM. While these are limitations of this study design, the following mitigating factors should be noted. First, long-listing of symptoms is typically restricted to interviews and the involvement of a large survey group (n = 224) is an additional extension. Second, all interview outcomes were widely represented in the survey, and no female selected the gender-specific erectile dysfunction, indicating internal consistency. Third, sampling demographics across outcomes was consistent with the overall survey, and aside the noted female predominance, those who had undergone surgery shared demographics with the leading prospective series from the literature. Finally, the use of the internet has enabled an efficient and broad reach of DCM, including a large sample of pre-surgical DCM, poorly represented in the conventional literature.^18,25^ Internet recruitment has been a mainstay of core-outcome setting processes, with no additional validation.^27^

It should also be noted that a number of included outcomes were taken from the open-ended survey question. While each of these included multiple references (Table 3), they have not undergone wider assessment. For clarity, these have been marked as red in Tables 2 and 3.

As outlined, the principal aim of this study was to develop a long list of outcomes from the perspective of those living with DCM, in order to inform the AO Spine RECODE-DCM COS.^17^ The findings of this study will be supplemented with findings from literature reviews^5,28^ and refined using an online, international DELPHI survey and finally a face to face consensus meeting.

### Findings in Context

Most of the reported outcomes can fit within our conventional understanding of DCM, albeit they are rarely assessed as part of clinical research^5^ or care.^29^ However, others less so.

This includes female sexual dysfunction, which unlike erectile dysfunction, is not routinely evaluated.^29^ This likely relates to it being an indirect consequence of DCM; one respondent wrote *Unable to orgasm—lack of sensation* (Supporting Information 4, see Supplementary Material). The importance of sexual dysfunction to quality of life in traumatic spinal cord injury was evidenced by Kim Anderson,^30^ but notably this was largely among young male sufferers. The prevalence in DCM needs to be explored among a larger population before firm conclusions are drawn.

Likewise, sensation, which was frequently referenced by PwCM, including a much broader breakdown of its perception and location than is routinely considered: the international standard for disease severity, the mJOA, restricts this to none, mild, moderate, or severe “numbness” in the hands only.^22^ While more sophisticated tools exist, none would assess all of these domains or locations entirely.^3^ Clearly some pragmatism needs to be applied to clinical assessments and it is worth noting that PwCM prioritize recovery of pain far beyond other domains of sensation, indicating there are important differences to consider here.^20^

Our experience of traumatic spinal cord injury indicates the potential for both respiratory, cardiovascular, gastrointestinal, and involuntary movement outcomes.^9,31^ The striking feature here is their purported prevalence in DCM, among a population in whom this is not typically considered; 19% to 41% survey respondents reported breathing difficulties and 38% to 66% involuntary movement disorders. Recent studies have demonstrated quantitative respiratory^10,32^ and cardiovascular dysfunction,^33^ which responds to DCM surgery. However, typically these descriptions are of subclinical findings. The experience reported by PwCM here is clearly conscious. It is worth noting a screening questionnaire developed in a neurosurgical clinic in Japan for myelopathy found the presence of chest tightness specific.^12^

Another prevalent and unexpected feature reported was variability, with its logical impact on social planning and enjoyment. This was reported by 66% of sufferers, with 42% reporting it could change hour-by-hour. The emerging experience from assessment and management of mild myelopathy does indicate that there can be an adaptation, with perhaps slight improvement in disability without treatment in the short term. The role of spinal cord hypoperfusion in the etiology of DCM is also of interest here,^34^ with the mention of activity dependent symptoms.

While referenced by numerous individuals, the “less known” symptoms were not validated across the whole surveyed population, instead submitted as additional suggestions by respondents. In the literature, some of these “less known” symptoms are labeled as “Barré-Liéou Syndrome.” The famous French neurologist, who also described Guillain-Barré syndrome, described a constellation of symptoms secondary to altered sympathetic transmission as a result of cervical spondylosis.^35^ The evidence base for this is low quality, and largely predates 1990. In the context of surgical treatment, most recent research has stemmed from groups in China, who describe symptom response with surgical treatment.^26,36^ It should be noted components of this syndrome, including cervicogenic headache^37,38^ and cervicogenic dizziness,^36^ have their own individualized research base, including ICD (International Classification of Diseases) codes.

However, these associations remain controversial and it should be noted that these series largely focus on cervical spondylosis and not myelopathy. The link to myelopathy specifically may therefore be even more tentative.

We recently reported on an unusual case of sensory dysesthesia in DCM involving the body but also the face.^39^ Our prevailing clinical view was that the facial symptoms represented a psychosomatic overlay but conceptualized that projections of the nucleus-tractus solitarius do project into the cervical spinal cord and altered trigeminal nerve processing was theoretically possible. Additionally, the emerging evidence of structural cerebral re-organization in response to myelopathy^40,41^ questions whether there is associated altered central processing. Of note, Chen et al^42^ and Takenaka et al^43^ using functional magnetic resonance imaging to investigate pre- and postsurgical connectivity changes in the brain have both, independently, identified changes in the visual cortex able to distinguish DCM from healthy controls, and correlating with surgical outcome. While we therefore remain skeptical, as popularized by Carl Sagan, *An absence of evidence is not evidence of absence*. So, if the burden is truly prevalent and significant among our population, regardless of exact etiology, it warrants further consideration.

## Conclusions

This study provides the first, comprehensive list of outcomes associated with DCM from the perspective of people living with the condition. Many reported outcomes are not currently evaluated in clinical research or care. While many can be reconciled within conventional understanding, many will be controversial.

## Supplementary Materials

Supplemental Material, Supplementary_Data.b - Outcomes of Degenerative Cervical Myelopathy From The Perspective of Persons Living With the Condition: Findings of a Semistructured Interview Process With Partnered Internet Survey Click here for additional data file. Supplemental Material, Supplementary_Data.b for Outcomes of Degenerative Cervical Myelopathy From The Perspective of Persons Living With the Condition: Findings of a Semistructured Interview Process With Partnered Internet Survey by Benjamin M. Davies, Colin Munro, Danyal Z. Khan, Siobhan M. Fitzpatrick, Bryn Hilton, Oliver D. Mowforth, Angus G. K. McNair, Iwan Sadler and Mark R. N. Kotter in Global Spine Journal
