# Supplementary material for: Outcomes of Degenerative Cervical Myelopathy From The Perspective of Persons Living With the Condition: Findings of a Semistructured Interview Process With Partnered Internet Survey
Source: Global Spine J. 2020 Nov 18;12(3):432–40. doi: 10.1177/2192568220953811 (PMC9121154; doi:10.1177/2192568220953811)
Supplement: Supplemental Material, Supplementary_Data.b - Outcomes of Degenerative Cervical Myelopathy From The Perspective of Persons Living With the Condition: Findings of a Semistructured Interview Process With Partnered Internet Survey [file Supplementary_Data.b.docx]

# Supplementary Data

Supporting Information 1*:* Carer and Patient reported effects of DCM. PwCM identified more effects than Carers.

| **Patient Reported Effects of DCM, from interviews** | **Carer Reported Effects of DCM, from interviews** |
| --- | --- |
| Balance  Falls  Lack of control of legs  Dragging Legs  Heavy Legs  Leg Shaking  Shaking  Erectile Dysfunction  Incontinence  Side effects of medication  Secondary disease, such as obesity  Lower Back Pain  Hip Pain, from walking stick  Dependence  Difficulty travelling around  Anxiety  Social Stigma  Feeling fobbed off  Reduced quality of life  Loss of concentration  Pins and Needles  Fear  Guilt for others around them  Frustration  ‘But you look normal’  Pay back fatigue  Financial  Unemployment  Grip Strength  Hand Shaking  Loss of dexterity  Muscle Spasms  Unable to exercise  Reduced activity  Difficulty climbing stairs  Reduced walking distance  Inability to drive  Weakness  Can’t get up  Payback pain  Neck Pain  Restless legs / spasms  Stiffness  Neck Clicking  Burning pain  Numbness  Sweating  Loss of temperature regulation  Breathing difficulties  Insomnia  Unable to sit comfortably  Unable to roll over in bed  Unpredictability  Variability | Incontinence  Loss of bladder control  Difficulty starting to urinate  Leg Pain  Difficulty doing up buttons  Arm Shaking  Reduced dexterity  Side effects of medication  Lack of coordination  Depression  Anxiety  Social Consequences  Financial Consequences  Altered cognition  Unpredictability  Difficulty sleeping  Pain free day  Unable to make plans |

Supporting Information 2: The unique effects identified through patient and carer interviews were combined, categorised according to theme, and adapted by authors BMD, CM and IS for external validation in an internet survey. Authors referred back to audio-recording to support adaptation. Adaptation included adding detail (e.g. the location of a symptom arm and/or leg) and changing the wording for clarity (adding reduced to ‘grip strength’). However given the initial list of outcomes was formed by PwCM and carers, the default position was to continue with their original wording. Secondary consequences (E.g. ‘ Hip Pain, from walking stick’) were considered out of scope for this initative and removed.

| **Interview Identified Outcomes (Categorised by Authors)** | **Interview Identified Outcomes, Processed for Internet Survey** |
| --- | --- |
| Balance  Falls  Lack of control of legs  Dragging Legs  Heavy Legs  Leg Shaking  Difficulty climbing stairs  Reduced walking distance  Weakness  Grip Strength  Hand Shaking  Loss of dexterity  Shaking  Muscle Spasms  Lack of coordination  Erectile Dysfunction  Incontinence  Difficulty starting to urinate  Side effects of medication  Secondary disease, such as obesity  Lower Back Pain  Hip Pain, from walking stick  Dependence  Difficulty travelling around  Inability to drive  Unable to exercise  Reduced activity  Can’t get up  Anxiety  Fear  Frustration  Social Stigma  Feeling fobbed off  ‘But you look normal’  Reduced quality of life  Loss of concentration  Altered cognition  Guilt for others around them  Pay back fatigue  Financial  Unemployment  Payback pain  Neck Pain  Restless legs / spasms  Stiffness  Neck Clicking  Burning pain  Numbness  Pins and Needles  Sweating  Loss of temperature regulation  Breathing difficulties  Insomnia  Unable to sit comfortably  Unable to roll over in bed  Unpredictability  Variability  Unable to make plans | Hand shaking  Reduced grip  Reduced dexterity (less able to perform complex tasks with your hands)  Muscle spasms or twitches (in your arms)  Leg shaking  Heavy legs  Dragging legs  Muscle spasms or twitches (in your legs)  Imbalance  Lack of control of legs  Clumsiness  Difficulty emptying bladder  Urinary incontinence  Faecal incontinence  Erectile  Symptom variability day by day  Symptom variability hour by  Insomnia  Waking to go to the toilet  Difficulty breathing when performing physical activity  Difficulty breathing when lying flat  Hot flushes and/or sweating  Numbness  Pins and needles  Neck pain  Arm pain  Leg pain  Back pain  Neck stiffness  Arm stiffness  Leg stiffness  Neck clicking  Depression/low mood  Anxiety  Impaired cognition  Fatigue  Falls  Unable to drive  Unable to exercise  Difficulties climbing stairs  Unable to get up out of a chair  Unable to get out of bed  Muscle weakness  Reduced walking distance  Reduced activity such that you are unable to have fun  Reduced activity such that you are unable to work  Difficulty with travel  Difficulty with planning life  Inability to turn over in bed  Unable to get comfortable in bed  Difficulties with social interaction  Financial difficulties |

Supporting Information 3: Additional suggestions, not already listed, from the first round survey. These were reviewed by authors BMD, CM and IS and coded as ‘out of scope’ (blue-strike through), ‘already included’ (green-strike through) and ‘new’ (black). A blank line indicates a new survey submission. New suggestions were then processed and incorporated into the outcomes list by mutual agreement.

| **Additional Symptoms, submitted via survey** | **Additional Life Effects, submitted via survey** |
| --- | --- |
| ~~Swollen Hands~~, Nausea.  Dizziness  ~~Progressive numbness of feet & legs, followed by hands & arms. Progressive weakness and loss of function of arms.~~  Headache, ~~can't sleep, balance, bending over causes neck/head pain, can't stay in one position long, feet feel like I have socks on,~~ skin burning sensation light touch numbness over surface of whole body, shower hurts like needles  ~~Ability to focus / Drowsiness~~  ~~Cramping of hands and fingers into distorted shapes and cramping of toes.~~ Choking a lot when eating food.  ~~Weakness in arms,legs and hands~~  ~~Dizziness~~  Shoulder pain v early symptom  ~~Tingling alone~~  ~~Heightened reflexes and night cramps~~  ~~Sexual difficulty~~  bladder/abdominal pain, kidney pain, headaches, burning feet hands  ~~Numbness in feet~~  muscle tightness on right side of head and face. caused significant eye pain prior to surgery. now just tightness  Eyesight, tension/pressure at the top and back of head  ~~Muscle atrophy~~  ~~loss of temperature and pain sensations with burning skin sensation on left side of the body and loss of strength on the right side of the body~~  Headaches  ? Swallowing problem.  Lack of sexual function.  Dizzyness, Constipation,  Dizziness, Numbness in face, Headache  ~~Staggering triggers driving more than 45 mins wearing heels more than 2 inch~~  Heat and cold intolerance, difficulty regulating temperature, headaches, face pain, allodynia,  Possible eye site fluctuating in vision  ~~Delay beginning urination. Drop foot. Clonus.~~  ~~My balance was badly affected and some days still is~~  Hypersensitive right leg, electric shock sensations in hands and feet with bending before surgery  My hands would fall off the steering wheel while driving, vertigo and tunnel vision  ~~My scapula was involved as well as muscle spasms all the way to my buttocks~~  ~~Poor balance, wobbliness~~  ~~Instability when walking slowly/ standing~~  ~~Have to use catheter 2/3times a day~~  Chronic Headaches  ~~Chills. Muscle spasms in ribs cage. Foot drop. Wrist pain. Loss of muscle tone in hands. And arms~~. Tinnitus  ~~Loss of balance , gait~~  Unable to pass stools unaided need medication  Constipation, ~~spasms, balance problems,~~ heavy head. Numbing of face, acute hearing, painful outer ear, abdominal pain and bloating, dizziness, head rush. burning pain in neck  Dizziness when looking up, down and turning in bed. Fogginess somedays; a feeling of disconnectedness.  Arrhythmia due to lying down  Burning on both hands  ~~Loss of balance~~  ~~Burning sensation in lower legs and impaired muscle tone in lower legs and feet.~~  ~~Tingles around the body,~~ dizziness, mood swings  Random bouts of nausea/vomiting  ~~Loss of balance, foot and lower leg cramps, shoulder tip pain~~  ~~Trying to claim disability is horrendous and has affected my illness despite evidence~~  ~~Lock jaw, cannot move eyes up and down side to side. Sometimes cannot open my eye lids. Only if I move neck left or right symptoms go.~~  I experienced a feeling of being stabbed by a knife in arm  ~~High arches and pain in feet. Muscle atrophy left shoulder~~  Burning/freezing/numbness in feet, sharp pain in ear, neuralgic pain that moves to various locations in body. Face feels as if it has been wind burned/chapped.  ~~Complete loss of left arm, usage.~~  ~~Loss of balance~~  ~~Loss of heat and pain sensation on the left half of my body and loss of strength on the right half~~  Female sexual problems. ~~Loss of feeling and sensation in genital area. Shoulder pain and numbness, numb patches in torso~~  ~~Headaches!!~~  ~~Fibromyalgia, Hashimoto's, DDD.~~  Severe neuropathic pain in all four limbs and trunk. It can feel like electricity beginning on the back bottom of my neck and deep in my shoulder blades, and the pain shocks clear through my under arms to my fingers, as well as down my back, buttocks, feet and toes. I can only imagine that is what it would feel like to be struck by lightning.  Severe itching in fingers  Spatial/ sensory/ proprioception issues  Female sexual dysfunction  ~~Foot cramping, spasms~~  ~~Hot and cold patches on skin~~  Headrush, sensitive hearing, head pain, itchy/burning skin  I may feel like I'm treading water.  Stabbing, burning pain in random places and for no apparent reason. May last a second or minutes. Mainly in feet toes and hands. ~~Wasted thenar muscles.~~  ~~Very poor balance (first symptoms)~~  Running shocks down arms to hands, duzziness  Also have Myelomalacia. Lose balance, autonomic dysfunction, including dizziness, orthostatic hypotension, inability to control body temperature and overdilation of veins, pain in hands and feet, swelling and burning in all extremities and face, headaches  Severe headaches, ~~extreme weakness,~~ ~~crying for no reason~~  ~~Total loss of the use of my left arm. Whole arm.~~  ~~Finger spasms~~  Reduced range of movement in neck and spine, ~~muscle wasting in hands,arms,legs and feet~~  ~~Tics & twitches that occur randomly, without warning, usually not painful. Also legs/ankles giving way without warning, although not always falling as a consequence. Feelings of discomfort in presence of someone tapping their foot on the floor or on the back of the sofa - uncomfortable. Symptoms vary hour to hour too. Difficulty climbing hills or walking up stairs~~  ~~Emptying bladder~~ | ~~Panic attacks~~  Unpredictability  ~~I had a pain in my hand and numbness, tingling in my legs just after I was diagnosed to present day~~  parenting ~~and work are~~ extremely difficult  Eye Sight - Bad Blurred Vision ~~/ Inability to absorb Iron Feritan levels always low~~  ~~Had to change job and reduce hours early on~~  ~~Feeling of failure~~  ~~Loss of friends, intimacy, joy~~  ~~I can drive but only short distances.~~  ~~Some of the above effects are caused by lumbar DDD~~  Vision problems  ~~Difficulty turning in bed~~  ~~Lifting Heavy Things~~  ~~Feeling of isolation no one even professional colleagues seem aware of this condition or what I deal with on a daily basis I am still working (Occupational Health Advisor)~~  Reduced sex life  ~~Since surgery unable to scuba dive, a hobby that I was passionate about and dived regularly. This is due to the medication I'm on for pain relief.~~  I find it difficult to decide what to do some times  ~~Change in employment due to condition~~  ~~Chronic pain is very disruptive to your social life and all relationships~~  ~~Unable to concentrate and organise things~~  Feeling might lose consciousness if turn over quickly in bed  ~~Intermittent loss of bowl control~~  Cannot lay flat hence sleep in riser recliner chair  ~~Feel isolated. Balance problems make bus, train and flying very difficult.~~  Difficulty concentrating and focusing, using the wrong words, saying words in wrong order, forgetting words and names of everyday things, forgetting processes like looking at the microwave and not knowing how to use it momentarily  Serious effect in as much I lost the last year's of my two youngest sons teenage lives in as much as I was unable to join them in outdoor activities which we did as a family which in turn added to my already depressed moods regarding my much changed capabilities  Can drive, not long distance or times  Going down stairs. Hiking or walking on uneven ground. Nature of activity and travel impacted by medical advice to wear hard collar.  Unable to stand for long, difficulties prep meals, need mobility scooter  ~~Myoclonus,~~ ~~seizures~~, migraine-like headaches  ~~Unable to run or increase walking pace or speed.~~  ~~Lack of sleep causing fatigue and day to day tiredness.~~  Trying to claim disability has led to decline through stress  ~~I can go from full paralysis to a greyhound out of a trap. It’s the position of my neck. Over the years I have found out how to have better mobility. The worst time for me is relaxing in a chair, that is when I can go into total paralysis with bladder retention.~~  ~~I can only drive when I feel like I can control a car. I live alone, or else I would never drive a car.~~  ~~Unable to work - too dangerous with risk of falls & potential paralysis~~  ~~Driving is becoming a problem. Doing day to day activities are becoming more and more tiring.~~  ~~Lack of understanding from others including DWP!~~  ~~Doctor retired me early, on Disability Pension~~. Can't walk up or down stairs, or ramps, in constant Vertigo sensation~~. Can't walk longer than about 40 mins. Otherwise body feeling goes and have no control over legs to keep walking.~~  Unable to fulfill basic household duties, cleaning, dishes, laundry, etc. Unable to fulfill parenting duties, taking kids out, preparing meals, playing at home.  ~~Difficulty driving due to arm pain, tightness, and spasms.~~  Fear of further damage due to work/actvity  Unable to bend or reach up, unable to stir, raise arms - everything becomes a challenge, it's exhausting  Sexual problems. Numbness and loss of feeling in genital area in the early months. Unable to reach orgasm ( Lack of sensation).  Unable to drive due to pain meds, not the illness itself.  Fear of paralysis, ~~inability to cook meals, deal with household chores, gardening etc~~  ~~Unable to get up off the floor without help or support of e.g. furniture after a fall. Loss of balance if move too quickly~~ |

Supporting Information 4: Demographics for those reporting an individual outcome from the internet survey.

| **Outcome** | **Prevalence (%)** | **For those experiencing an outcome** | | | | | |
| --- | --- | --- | --- | --- | --- | --- | --- |
|  |  | **mJOA (±SD)** | | **Female (%)** | **Undergone Surgery (%)** | **Time lived with DCM (Years ± SD)** | |
| Hand shaking | 42% | 10.4 | 2.7 | 79% | 64% | 9.1 | 9.5 |
| Reduced grip strength | 81% | 11.3 | 2.6 | 80% | 64% | 8.4 | 9.0 |
| Reduced dexterity | 76% | 11.0 | 2.6 | 76% | 65% | 8.6 | 9.2 |
| Muscle spasms or twitches (in your arms) | 62% | 11.2 | 2.5 | 78% | 67% | 8.2 | 8.9 |
| Leg shaking | 38% | 10.7 | 2.4 | 74% | 69% | 8.6 | 9.2 |
| Heavy legs | 71% | 11.1 | 2.5 | 75% | 65% | 8.2 | 8.9 |
| Dragging legs | 50% | 10.4 | 2.4 | 74% | 69% | 8.5 | 8.7 |
| Muscle spasms or twitches (in your legs) | 66% | 11.0 | 2.4 | 78% | 65% | 8.5 | 9.0 |
| Imbalance | 43% | 10.1 | 2.4 | 77% | 65% | 10.7 | 10.6 |
| Lack of control of legs | 31% | 10.1 | 2.1 | 75% | 75% | 9.6 | 10.3 |
| Clumsiness | 75% | 10.9 | 2.4 | 76% | 67% | 9.0 | 9.3 |
| Difficulty emptying bladder | 41% | 10.7 | 2.7 | 77% | 60% | 8.6 | 8.2 |
| Urinary incontinence | 37% | 10.6 | 2.3 | 83% | 65% | 8.4 | 7.4 |
| Faecal incontinence | 12% | 10.8 | 2.8 | 65% | 69% | 6.8 | 6.2 |
| Erectile Dysfunction | 34% | 9.9 | 2.7 | 0% | 62% | 9.8 | 13.3 |
| Symptom variability day by day | 66% | 11.5 | 2.6 | 79% | 62% | 8.8 | 9.8 |
| Symptom variability hour by hour | 42% | 11.0 | 2.4 | 77% | 63% | 9.1 | 10.1 |
| Insomnia | 56% | 11.2 | 2.7 | 82% | 64% | 8.6 | 9.4 |
| Waking to go to the toilet | 53% | 11.4 | 2.6 | 76% | 64% | 8.4 | 9.0 |
| Difficulty breathing when performing physical activity | 41% | 10.8 | 2.4 | 77% | 64% | 8.4 | 8.2 |
| Difficulty breathing when lying flat | 19% | 10.5 | 2.4 | 69% | 57% | 8.5 | 9.7 |
| Hot flushes and/or sweating | 55% | 11.3 | 2.8 | 87% | 65% | 9.2 | 9.9 |
| Numbness | 80% | 11.2 | 2.6 | 78% | 65% | 8.2 | 8.6 |
| Pins and needles | 80% | 11.5 | 2.9 | 81% | 64% | 8.1 | 8.6 |
| Neck pain | 78% | 11.5 | 2.9 | 83% | 61% | 8.7 | 9.3 |
| Arm pain | 73% | 11.3 | 2.7 | 79% | 65% | 9.0 | 9.5 |
| Leg pain | 60% | 10.7 | 2.5 | 84% | 64% | 9.6 | 9.8 |
| Back pain | 65% | 11.2 | 2.7 | 83% | 65% | 9.2 | 9.8 |
| Neck stiffness | 77% | 11.5 | 2.8 | 78% | 63% | 8.6 | 8.9 |
| Arm stiffness | 49% | 10.8 | 2.6 | 79% | 64% | 8.9 | 8.8 |
| Leg stiffness | 51% | 10.6 | 2.4 | 82% | 70% | 9.5 | 9.5 |
| Neck clicking | 63% | 11.5 | 2.8 | 81% | 64% | 9.3 | 9.6 |
| Depression/low mood | 66% | 11.4 | 2.9 | 79% | 63% | 8.6 | 9.3 |
| Anxiety | 62% | 11.2 | 3.0 | 78% | 63% | 8.9 | 9.2 |
| Impaired cognition | 46% | 10.7 | 2.6 | 78% | 67% | 9.7 | 10.3 |
| Fatigue | 83% | 11.4 | 2.7 | 80% | 65% | 8.5 | 9.2 |
| Falls | 38% | 9.7 | 2.2 | 77% | 67% | 9.6 | 9.5 |
| Unable to drive | 25% | 9.9 | 2.5 | 82% | 63% | 8.9 | 12.0 |
| Unable to exercise | 54% | 10.8 | 2.5 | 79% | 63% | 9.6 | 10.1 |
| Difficulties climbing stairs | 64% | 10.6 | 2.3 | 74% | 65% | 9.2 | 9.7 |
| Unable to get up out of a chair | 25% | 9.5 | 2.2 | 73% | 67% | 11.8 | 12.5 |
| Unable to get out of bed | 16% | 9.2 | 2.4 | 69% | 72% | 10.5 | 9.6 |
| Muscle weakness | 76% | 11.2 | 2.7 | 76% | 65% | 9.0 | 9.3 |
| Reduced walking distance | 70% | 10.7 | 2.4 | 75% | 64% | 8.7 | 8.4 |
| Reduced activity such that you are unable to have fun | 71% | 11.0 | 2.7 | 78% | 62% | 8.9 | 9.5 |
| Reduced activity such that you are unable to work | 53% | 10.6 | 2.6 | 76% | 64% | 8.9 | 9.8 |
| Difficulty with travel | 60% | 10.5 | 2.5 | 77% | 69% | 9.4 | 10.0 |
| Difficulty with planning life | 54% | 10.7 | 2.7 | 76% | 64% | 8.4 | 8.2 |
| Inability to turn over in bed | 26% | 10.4 | 2.7 | 86% | 61% | 10.3 | 11.5 |
| Unable to get comfortable in bed | 62% | 11.1 | 2.8 | 83% | 62% | 8.5 | 9.6 |
| Difficulties with social interaction | 47% | 10.1 | 2.5 | 79% | 62% | 9.0 | 9.8 |
| Financial difficulties | 47% | 10.5 | 2.4 | 78% | 64% | 9.1 | 9.8 |
